# Evaluation of the Mycobactericidal Effect of Thio-functionalized Carbohydrate Derivatives

**DOI:** 10.3390/molecules22050812

**Published:** 2017-05-16

**Authors:** Małgorzata Korycka-Machała, Anna Brzostek, Bożena Dziadek, Malwina Kawka, Tomasz Popławski, Zbigniew J. Witczak, Jarosław Dziadek

**Affiliations:** 1 Institute of Medical Biology, Polish Academy of Sciences, Lodz 93-232, Poland; mkorycka@cbm.pan.pl (M.K.-M.); abrzostek@cbm.pan.pl (A.B.); 2 Department of Immunoparasitology, University of Lodz, Lodz 90-236, Poland; bozena.dziadek@biol.uni.lodz.pl (B.D.); malwina.kawka@gmail.com (M.K.); 3 Department of Molecular Genetics, University of Lodz, Lodz 90-236, Poland; tomasz.poplawski@biol.uni.lodz.pl; 4 Department of Pharmaceutical Sciences, Nesbitt School of Pharmacy, Wilkes University, Wilkes-Barre, PA 18766, USA

**Keywords:** thiosaccharides, *Mycobacterium tuberculosis*, bactericidal effect

## Abstract

Sugars with heteroatoms other than oxygen have attained considerable importance in glycobiology and in drug design since they are often more stable in blood plasma due to their resistance to enzymes, such as glycosidases, phosphorylases and glycosyltransferases. The replacement of oxygen atoms in sugars with sulfur forms thio-sugars, which are potentially useful for the treatment of diabetes and some bacterial and viral infections. Here, we evaluated the antibacterial activity of thio-functionalized carbohydrate derivatives. A set of 21 compounds was screened against acid-fast *Mycobacterium tuberculosis* (*Mtb*), gram-negative *Escherichia coli* and gram-positive *Staphylococcus aureus.* The tested carbohydrate derivatives were most effective against tubercle bacilli, with as many as five compounds (thioglycoside **6**, thiosemicarbazone **16A**, thiosemicarbazone **20**, aminothiadiazole **23**, and thiazoline **26**) inhibiting its growth with MIC_50_ ≤ 50 µM/CFU. Only two compounds (aminothiadiazole **23** and thiazoline **26**) were able to inhibit the growth of *E. coli* at concentrations below 1 mM, and one of them, aminothiadiazole **23**, inhibited the growth of *S. aureus* at a concentration ≤1 mM. The five compounds affecting the growth of mycobacteria were either thiodisaccharides (**6**, **16A**, and **20**) or thioglycosides (**23** and **26**). All of these compounds (**6**, **16A**, **20**, **23**, and **26**) were able to inhibit the growth of *Mtb* deposited within human macrophages. However, three of the five selected compounds (**6**, **23**, and **26**) exhibited relatively high cytotoxicity in mouse fibroblasts at micromolar concentrations. The selected thio-sugars are very promising compounds, thus making them candidates for further modifications that would decrease their cytotoxicity against eukaryotic cells without affecting their antimycobacterial potential.

## 1. Introduction

In the early 1980s, the first studies of carbohydrate-binding proteins prompted a new era of research and development into the roles of carbohydrates and carbohydrate-binding proteins in biological systems. Since then, numerous scientific studies have uncovered several important structure-activity relationships for carbohydrate-based compounds [1,2]. These studies have demonstrated that the diversity and complexity of carbohydrates permit them to carry out a wide range of biological functions. Research on carbohydrates is now experiencing considerable growth and promises to be a major source of drug-discovery leads. Carbohydrate-based drug development has emerged as a highly promising and exciting area in contemporary medicine and chemistry. Interest in this area is based on a number of factors, including our growing understanding of the role of endogenous carbohydrates in cellular function, our ability to synthesize carbohydrate analogues, and the success of some of these analogues as novel therapeutic agents for the treatment of cancer, diabetes, and infectious diseases [3,4,5]. As a result, there has been a significant increase in the number of reviews that address the general field of carbohydrate medicinal chemistry. These reviews are devoted to examining the new developments in carbohydrate drug design, with focus on the use of new functionalized carbohydrates as promising therapeutic agents [3,4,5]. All of the new strategies, whether currently available or under development, constitute significant steps in the area of carbohydrate therapeutics. Therefore, the development of carbohydrate-based drugs represents a novel approach to treating life-threatening disorders. So far, there are two examples of thio-sugars used as therapeutic agents: thiolactomycin [6] and tagetitoxin [7]. Tuberculosis, a common infectious bacterial disease, is the second leading cause of mortality due to a single infectious agent worldwide. Despite widely available medical treatments, tuberculosis remains a global pandemic, killing one person every 25 s. This mortality rate is a result of a lack of an effective tuberculosis vaccine as well as the pathogen’s emerging multidrug resistance and the vulnerability that HIV-TB co-infected patients exhibit towards it. Therefore, developing alternative medical strategies, new drugs and new targets for tuberculostatics are top priority areas of tuberculosis research. The new hope for TB treatment is novel drugs in preclinical development and several new compounds being assessed at all stages of clinical trials [8,9,10,11]. The enrichment of the anti-TB drug portfolio is being achieved through the repurposing of old drugs, re-engineering of existing antibacterial compounds and discovery of new compounds. The most advanced phase 3 studies being carried out are for bedaquiline and delamanid, which are conditionally approved for the treatment of multidrug resistant (MDR) and/or extensively drug resistant (XDR) tuberculosis [12]. Other drugs, such as efflux pump inhibitors, show antimycobacterial potency in preclinical studies [13,14,15]. The conventional antituberculosis drugs and their drug targets were described previously in a number of review papers [16,17] The selection of new anti-TB drugs that could satisfy all of the criteria of preclinical and clinical trials requires the discovery and screening of a huge number of anti-TB drugs candidates, including carbohydrate-derived compounds, which are the focus of this work. The hydrophilic/lipophilic balance of thiosugars and thiosemicarbazone moiety selected for this work make them potentially good candidates for new putative drugs.

## 2. Results

### 2.1. The Bactericidal Activity of Carbohydrates

A variety of carbohydrate-derived compounds were evaluated with respect to their activity against tubercle bacilli and control bacteria, Gram-negative *E. coli* and Gram-positive *S. aureus* (Table 1). All of the tested compounds were evaluated in vitro at concentrations between 1–0.25 mM against *M. tuberculosis* in susceptibility assays based on the optical density (OD_600_) of the growth culture. For active compounds, the MIC was determined by CFU analysis, as described in the methods section. The broth microdilution assay was applied with a maximum concentration of 10 mM to control the susceptibility of *E. coli* and *S. aureus.* The monosaccharides containing a hemi-thioacetal functionality in which the sulfur atom comprised part of the ring [5-thio-d-glucose (compound **1**) and 6-thio-d-fructopyranose (compound **2**)] were not bactericidal against *M. tuberculosis* or the control bacterial strains (Table 1). The inhibition of bacterial growth was also not observed for pyranose compounds featuring an anhydrosugar motif in which both oxygen atoms forming the acetal were part of the five-membered (anhydro) ring and one of these atoms was part of the pyranose ring as well (compound **3**). The exocyclic epoxide coupled to the inositol moiety (compound **4**) and the 1,5-glucitol featuring the methanesulfonamide group (compound **5**) were also not active against the tested bacteria. On the other hand, bactericidal activity against *M. tuberculosis* was observed from some of the (1-4)-*S*-thiodisaccharides. The strongest anti-*Mtb* activity (MIC_50_ = 50 µM/CFU) was identified for 1,6-anhydro-3-deoxy-4-*S*-(2,3,4,6-tetra-*O*-acetyl-β-d-glucopyranosyl-d-glycerohexopyranos-2-ulose (**6**). Compound 7, a deacetylated analogue without the tetra-*O*-acetyl groups, required a 10-times higher concentration to inhibit the growth of *Mtb.* The replacement of the glucose moiety in compound **6** with galactose in compound **9** decreased the bactericidal activity at least 3 times. The replacement of 2,3,4,6-tetra-*O*-acetyl-β-d-glucopyranosyl with a salicylyl group (compound **8**) increased the MIC_50_ value for *Mtb* 4 times. A similar effect was observed when the glucopyranosyl moiety of compound **6** was replaced with a lactosyl moiety (compound **16**): it increased the MIC_50_ value for *Mtb* 10 times. However, the modification of compound **16** by replacing the keto group with a thiosemicarbazone significantly increased the bactericidal activity against *Mtb* (MIC_50_ = 30 µM/CFU) and made the compound active against *E. coli* (MIC_50_ = 150 µM/CFU). On the other hand, the bactericidal effect was completely lost when a thiosemicarbazone moiety replaced the keto group (compound **22**) in compound **6**; however, the same modification along with deacetylation at position 6 (compound **20**) restored the bactericidal effect against both *Mtb* and *E. coli.* The replacement of the glycero-hexopyranos-2-ulose moiety of compound 16 with an adamantane functionality (compound **19**) did not result in any change in bactericidal activity. The thiosemicarbazone present in the compound **14** did not significantly affect its bactericidal activity. We did not observe any bactericidal activity of the tested derivatives of mannitol, glucofuranose or pyranose (compounds **10**–**13**). The most significant anti-*Mtb* activity was identified from compounds carrying the 4,5-dihydrothiazole functional group (compound **26**) or the amino-thiadiazole group (compound **23**). Both compounds, **23** and **26**, appeared to be active against tubercle bacilli at concentration of 25 and 50 µM, respectively.

### 2.2. The Bactericidal Effect of Carbohydrates against Intracellularly Growing Tubercle Bacilli

**Figure 1:**
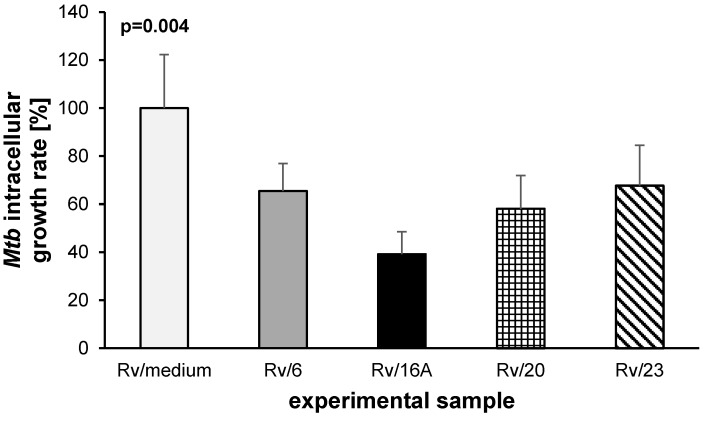
Inhibitory effects of carbohydrates **6**, **16A**, **20** and **23** on the intracellular growth of *Mycobacterium tuberculosis* in human monocyte-derived macrophages. The intracellular growth of the pathogen in the experimental (carbohydrate-treated) samples versus the control (carbohydrate-untreated) sample is presented as a growth rate, which represents the ratio of the CFU number determined for the carbohydrate-treated sample to the CFU number estimated in the control sample multiplied by 100%.

Tubercle bacilli are able to grow inside the macrophages of the host and are protected against drugs that are unable to penetrate inside human cells. Therefore, the activity of the putative anti-*Mtb* drugs should be evaluated against *Mtb*-infected macrophages. Four of the most effective compounds (**6**, **16A**, **20**, **23**) at inhibiting *Mtb* growth in broth were tested with respect to their bactericidal effects against tubercle bacilli growing inside human macrophages. The monocyte-derived macrophages were differentiated from peripheral blood mononuclear cells isolated from the buffy coats of healthy human blood donors. The compounds were used at the minimal concentrations that inhibited the growth of *Mtb* in broth culture: 50, 30, 30, and 25 µM for compounds **6**, **16A**, **20** and **23**, respectively. The same concentrations of compounds were used to evaluate the cytotoxicity against human phagocytes derived from the same blood donor. The incubation of macrophages in the presence of tested compounds for 48 h did not affect the viability of cells. The control phagocytes (no compound) and cells growing in the presence of each compound displayed over 91% viability, as detected by MTT assay (Table 2). Next, the monocyte-derived macrophages of a single donor were infected with *Mtb* at an MOI of 1:10. The bacteria were left undisturbed for 2 h for phagocytosis, then the extracellular bacilli were removed by intensive washing. Furthermore, the *Mtb*-infected cells were exposed to compounds **6**, **16A**, **20** and **23** and incubated for 48 h. The number of intracellularly located live bacteria was calculated by CFU. The data analysis showed the statistically significant (*p* = 0.004) reduction of viable bacilli isolated from cells incubated in the presence of the tested compounds (Figure 1), with the most substantial effect provided by compound **16A** (*p* < 0.001). In these samples, the number of intracellularly growing mycobacteria was decreased by almost 61%. Moreover, the analysis revealed that the other three functionalized carbohydrates tested, namely, **6** (*p* = 0.02), **20** (*p* = 0.005), and **23** (*p* = 0.03), were also able to significantly inhibit the growth of the intracellularly deposited tubercle bacilli.

### 2.3. The Carbohydrates’ Cytotoxicity

All carbohydrates used in this study were tested with respect to their cytotoxicity using a mice fibroblast cell line (Table 3). The IC_50_ value was calculated based on a colorimetric assay using the CCK-8 Kit and BioDataFit software. A significant cytotoxic effect, with IC_50_ = 4 µM, 11 µM and 45 µM, was observed for compounds **23**, **26** and **6**, respectively. The cytotoxicity of the other carbohydrates, which was calculated in a range of 0.1–1 mM, was 240 µM and 500 µM for compounds **16A** and **20**, respectively.

## 3. Discussion

Carbohydrates are used as an energy source and are components of various biomolecules, including nucleic acids or glycoproteins. Sugars with heteroatoms other than oxygen have attained considerable importance in glycobiology and in drug design [5]. The oxygen atom in carbohydrates can be replaced by sulfur, nitrogen, selenium, phosphorus or carbon, forming thio-sugars, iminosugars, selenosugars, phosphosugars or carbasugars, respectively. The presence of sulfur in thio-sugars make them resistant to glycosidases, phosphorylases and glycosyltransferases. There have been some reports [18,19] showing the ability of thio-sugars to impede cancer cell growth. It was shown that functionalized thio-sugars have cytotoxic properties and therapeutic potential as anticancer agents [20]. Thio-sugars and iminosugars are potentially useful in the treatment of diabetes and some bacterial and viral infection diseases [5,21,22]. It was also recently shown that iminosugar derivatives were able to inhibit biofilm formation by *Pseudomonas aeruginosa* [23]. Iminosaccharides were also demonstrated as inhibitors of glycosidases and glycosyltransferases involved in the biosynthesis of glycan chains [24], as well as UDP-galactopyranose mutase and UDP-galactofuranose transferases [25] involved in the formation of galactofuranose units and production of Gal*f*-containing polysaccharides [26,27] present in the exopolysaccharides of several bacteria [28,29]. Iminosugars were also reported as inhibitors of UDP-Gal*f* transferase, an enzyme involved in *Mtb* cell wall biosynthesis [26]. The moderate anti-mycobacterial activity was reported for a series of β-arabino glycosyl sulfones with varying alkyl chain lengths which potentially inhibits cell wall biosynthesis by mimicking decaprenolphosphoarabinose (DPA) [30]. The mycobacterial cell wall biosynthesis was also targeted by the range of triazole-linked 1,6-oligomannoside with the highest activity against mycobacterial α-(1,6)-mannosyltransferase observed for hexa- and octamannoside [31]. A thiosugar derivative, albomycin produced by *Streptomyces* sp. has a thioribosyl nucleoside moiety linked to an iron-chelating siderophore through a serine residue [32]. Albomycin was identified as a potent inhibitor against *S. aureus* [33]. It can be actively taken up by a bacterium through its siderophore-dependent iron acquisition system and is further activated intracelularly by peptidases resulting in MIC as low as 8 nM [34,35]. A thiosugar derivative active against tubercle bacilli is thiolactomycin (TLM). TLM was shown to exhibit anti-mycobacterial activity by specifically inhibiting fatty acid and mycolic acid biosynthesis by targeting β-ketoacyl-acyl-carrier protein synthases KasA and KasB, the members of FAS2 (fatty acids synthases) system [36]. It was also reported that thiodisaccharides (e.g., 1-thio-β-d-galacto-pyranosyl-β-d-galactopyranoside) exhibited bactericidal activity inhibiting growth of *E. coli* [37]. The main limitation in the use of carbohydrates as drugs is their hydrophilicity and bioavailability. The biologically useful compounds should present hydrophilic/lipophilic balance LogP/cLogP at −3.22/0.44 which is the case of the majority of compounds used in this work. 

Here, we evaluated the antibacterial activity of thio-functionalized carbohydrate derivatives. A total of 21 compounds were screened against acid-fast *M. tuberculosis*, Gram-negative *E. coli* and Gram-positive *S. aureus.* The selected bacteria differed in the composition of their cell walls, resulting in their dissimilar permeability for various compounds [38]. The tested carbohydrate derivatives were most effective against tubercle bacilli, with as many as 5 compounds inhibiting its growth with MIC_50_ ≤ 50 µM/CFU. Only 2 compounds (**16A** and **20**) were able to inhibit the growth of *E. coli* at concentrations below 1 mM, and one of them (**23**) inhibited the growth of *S. aureus* at a concentration ≤1 mM. The different activities of the tested compounds against *M. tuberculosis* and the other bacteria was not surprising since many drugs effective against *E. coli* and/or *S. aureus* are not effective against tubercle bacilli, and some antitubercular drugs are not effective against other bacteria [16]. The five compounds affecting mycobacteria were either thiodisaccharides (**6**, **16A**, and **20**) or thioglycosides (**23**, **26**). All of these compounds (**6**, **16A**, **20**, **23**, and **26**) in concentration of MIC_50_ partially inhibited the growth of *Mtb*-infected macrophages and these concentrations did not affect the viability of human mononuclear cells. However three out of five selected compounds (**6**, **23**, and **26**) displayed cytotoxicity in mouse fibroblasts at micromolar concentrations. These compounds require modification that to decreases their cytotoxicity against eukaryotic cells without affecting their antimycobacterial activity. Two compounds (**16A** and **20**), which inhibited the growth of *Mtb* at a concentration of 30 µM and were active against mycobacteria deposited in macrophages with cytotoxicities at concentrations ≥240 µM, seem to be most interesting. These compounds should be further used as substrates for the synthesis of derivatives effecting the growth of tubercle bacilli in nanomolar concentrations. These two compounds were the only ones able to partially inhibit the growth of *E. coli*, although at concentrations 5–10-times higher than in the case of *Mtb*. The biological activity of compounds **16A** and **20** could be related to thiosemicarbazone-substituted anhydrosugar, despite this substitution not being enough to impart activity to compound **14A**, which revealed an *Mtb* growth inhibitory effect but at 10-times higher concentration–350 µM. The biological activities of thiosemicarbazones are considered to be due to their ability to form chelates with metals. The antimycobacterial activity of thiosemicarbazones are well known since 1940s when thioacetazone was used in the therapy of tuberculosis. More recently the antibacterial, antifungal, antiviral, antiamoebic, antimalarial and antitumor activity of a number of compounds carrying thiosemicarbazone moiety was extensively published [39,40,41]. The 1,6-anhydro ring of functionalized thiosemicarbazones of anhydrosugars **14**, **14A**, **16A** and **20** it fixes the conformation of the system and most importantly, sterically hinders the β-d-face of the molecule. Moreover, it is well known that the anhydro bridge eliminates the need for protecting groups at the anomeric carbon and C-6-OH. Another very important factor is that the pyranose ring in functionalized C-4 products (C-4-substitutes levoglucosans) is locked in the ^1^C_4_ conformation (preferential biological conformation), which strongly suggest potentially better biological activity. Anhydrosugars substituted with thiazole and amino-thiadiazole group (compounds **26** and **23**) could also be interesting with respect to their antimycobacterial activity; however, the cytotoxicity of these compounds must be reduced. The evaluation of the antibacterial activity of functionalized carbohydrates performed in this work, suggested the antimycobacterial potential of several (**6**, **16A**, **20**, **23**, **26**) of the tested thio-functionalized sugars.

## 4. Materials and Methods

### 4.1. Chemistry

Most chemicals were obtained from the Aldrich Chemical Company (St. Louis, MO, USA) and were used without further purification unless otherwise stated. 

### 4.2. The Synthesis and Analysis of Carbohydrates

**Figure 2:**
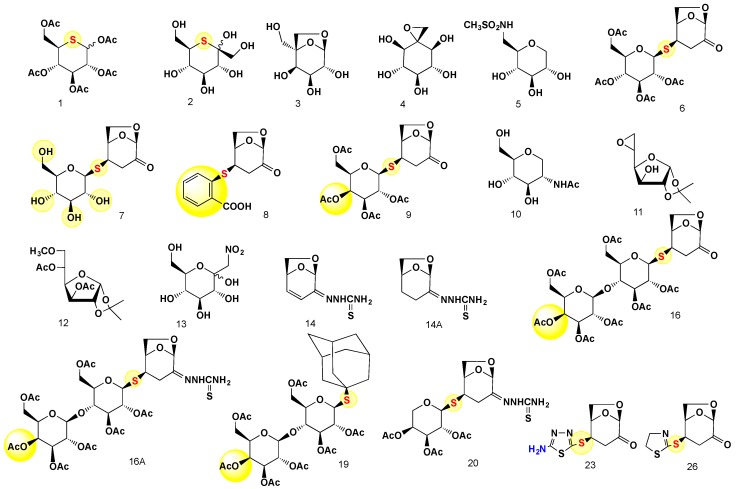
Thio-sugars, anhydrosugars and thio-functionalized disaccharides and their thiosemicarbazones evaluated in this study.

**Scheme 1:**
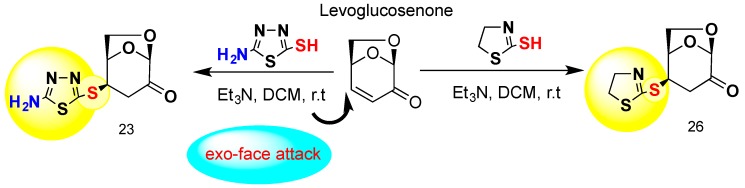
The stereoselective *exo*-face thiol attack addition at C-4 position with formation of 1,6-anhydro-deoxy-4-*S*-(5-amino-1,3,4-thiadiazol-2-yl)-d-glycero-*hexopyranos*-2-ulose (**23**) and 1,6-anhydro-3-deoxy-4-*S*-(4,5-dihydrothiazol-2-yl-d-glycero-*hexopyranos*-2-ulose) (**26**).

The carbohydrates evaluated in this study with respect to their antibacterial activity were synthesized as previously described (Table 4 and Figure 2) or prepared exclusively for this study (compounds **23** and **26**) (Table 4 and Scheme 1).

Compounds **23** and **26** were prepared exclusively for this work. The rationale for the synthesis of these compounds is related to the well-known pharmacological activity of two important pharmacophores, namely, 5-amino-1,3,4-thiadiazole and 4,5-dihydrothiazoline. Both of these heterocyclic pharmacophores are components of many pharmacologically active compounds [51,52,53]. The thiadiazole and dihydrothiazoline moieties are linked via a thio-bridge to the anhydro skeleton of levoglucosenone. Both *S*-glycosides, **23** and **26**, were prepared conventionally by our previously protocol of a thio-click Michael addition (TCMA) of reactive thiols to the conjugated system of levoglucosenone. We were able to demonstrate that exo-attack of two distinctively functionalized thiols are proceeding with identical stereoselective fashion with the formation of *S*-thioglycosides at the C-4 of the anhydrosugar moiety. The synthetic approach is depicted in Scheme 1.

The most important parameter of drug candidates is lipophilicity, which determines water solubility, absorption and often cytotoxicity. The lipophilicity of the synthesized compounds was measured as the partition (P) or distribution (D) coefficient. The octanol/water partition/distribution coefficient is a widely used parameter for measuring lipophilicity. The Log P value is the ratio of the concentrations of a neutral substance in two immiscible phases: water and octan-1-ol. Log D is the log partition at a particular pH, and it is usually reserved for partition coefficients measured under conditions in which the solute is partially or completely ionized in the aqueous phase. For all compounds used in this work, the LogP and cLogP values were determined (Table 4). Each compound was also analysed by TLC (thin layer chromatography) and ^1^H-NMR to verify its purity. All synthesized and tested compounds were analytically pure. Complete characterization of the products was performed by NMR spectroscopy and HR-MS techniques to confirm their purity and molecular structures.

### 4.3. General Information

The progress of all reactions was monitored by TLC on 2–5 cm precoated silica gel 60F_254_ plates with a thickness of 0.25 mm (Merck, Darmstadt, Germany). The chromatograms were visualized under UV (254–366 nm) and with iodine. NMR spectra were recorded on a PDX 400 instrument (Bruker, Billerica, MA, USA) in CDCl_3_. Chemical shift values are reported in δ (ppm) and coupling constants in Hertz. Mass spectra were recorded by ESI-MS 5975C Series GC/MSD (Agilent Technologies, Santa Clara, CA, USA). Melting points were recorded on a D-545 melting point apparatus (Büchi, New Castle, DE, USA) and are uncorrected. Chemical names were generated by ChemDraw Professional V.15.1.0.144 software (Perkin Elmer, Akron, OH, USA).

#### General Procedure for the Preparation of **23** and **26**

To a solution of levoglucosenone (1.26 g, 0.1 mmol) in dichloromethane (20 mL) was added a solution of the thiol (0.1 mmol) in 10 mL of dichloromethane, then triethylamine (0.5 mL) was added dropwise. The reaction mixture was stirred at room temperature for 24 h. After the evaporation of the solvent, the resulting syrupy residue was crystalized from ethanol to give pure crystalline products.

*(1R,2R,5R)-2-((5-amino-1,3,4-thiadiazol-2-yl)-thio)-6,8-dioxabicyclo[3.2.1]octan-4-one* (**23**), Yield (1.9 g, 73%), White solid crystals; m.p.: 140–142 °C; *R*_f_ = 0.74 (1:4, hexane–EtOAc); ${\left[\alpha \right]}_{D}^{30}$–124.3 (*c* 0.81, CHCl_3_); ^1^H-NMR: δ (400 MHz; CDCl_3_), 5.37 (d, 1H, H-1), 5.76 (d, *J*_3a-4_ = 9.6 Hz, 1H, H-4), 2.54 (d, 1H, H-3e), 4.92 (*d*, *J*_5-6*exo*_ = 5.5 Hz, 1H, H-5), 4.23 (d, *J*_gem_ = 8.2 Hz, 1H, H-6*endo*), 4.04 (dd, *J*_gem_ = 8.2 Hz, *J*_5-6exo_ = 5.5 Hz, 1H, H-6*exo*), 3.12 (dd, *J*_gem_ = 9.6 Hz, 1H, H-3a), 2.83 (d, *J*_gem_ =18.6 Hz, 1H, H-3b), 9.15 (s, 1H, -NH_2_); ^13^C-NMR δ (100 MHz; CDCl_3_) 31.2 (C-3), 68.4 (C-6), 70.6 (C-4), 78.2 (C-5), 104.4 (C-1), 112.1 (C-5, thiazole), 128. 9 (C-2, thiazole), 207.7 (C-2, C=O). HRMS *m*/*z* [M + H]: Calcd for C_8_H_9_N_3_O_3_S_2_: 259.30. Found: 260.0155. For copies of the ^1^H-NMR and ^13^C-NMR see Supplementary Materials.

*(1R,2R,5R)-2-((4,5-dihydrothiazol-2-yl)-thio)-6,8-dioxabicyclo[3.2.1]octan-4-one* (**26**), Yield (1.82 g, 74%), White solid crystals; m.p.: 127–129 °C; *R*_f_ = 0.57 (1:1, hexane–EtOAc); ${\left[\alpha \right]}_{D}^{30}$–134.2 (*c* 0.84, CHCl_3_); ^1^H-NMR: δ (400 MHz; CDCl_3_), 5.31 (d, 1H, H-1), 5.72 (d, *J*_3a-4_ = 9.4 Hz, 1H, H-4), 2.58 (d, 1H, H-3e), 4.92 (d, *J*_5-6*exo*_ = 5.3 Hz, 1H, H-5), 4.23 (d, *J*_gem_ = 8.4 Hz, 1H, H-6*endo*), 4.04 (dd, *J*_gem_ = 8.4 Hz, *J*_5-6exo_ = 5.5 Hz, 1H, H-6*exo*) 3.16 (dd, *J*_gem_ = 9.4 Hz, 1H, H-3a), 3.06 (d, *J*_gem_ = 16.9 Hz, 2H, C-4 thiazoline) 3.21(d, *J*_gem_ = 17.1 Hz, 2H, C-5, thiazoline), 2.81 (d, *J*_gem_ =18.4 Hz, 1H, H-3b); ^13^C-NMR δ (100 MHz; CDCl_3_) 31.1 (C-3), 36.7 (C-3, CH_2_-thiazoline), 39.4 (C-4, CH_2_-thiazoline), 68.4 (C-6), 70.6 (C-4), 78.2 (C-5), 104.4 (C-1), 128.7 (C-2, thiazoline), 207.2 (C-2, C=O). HRMS *m*/*z*, [M + H]: Calcd for C_9_H_11_NO_3_S_2_: 245.02, Found: 246.0252. For copies of the ^1^H-NMR and ^13^C-NMR see Supplementary Materials.

### 4.4. Bacterial Growth Conditions and Susceptibility Tests

The *Mycobacterium tuberculosis* H37Rv strain (*Mtb*) used in this study was cultured in Middlebrook 7H9 broth supplemented with 10% OADC (oleic acid-albumin-dextrose-catalase)) and 0.05% Tween-80 (pH 7) and given chemicals at various concentrations when required. The *M. tuberculosis* H37Rv strain was grown for 4–6 days until the optical density (OD_600_) reached 1. Then, the bacterial culture was suspended in fresh medium at an OD_600_ of approximately 0.1. The cultures were supplemented with the carbohydrates derivatives and grown for 96 h at 37 °C. The growth was monitored by OD_600_ and CFU analyses. The optical density (OD_600_) was monitored every 24 h for the control and carbohydrate-supplemented cultures. The number of viable bacilli was determined by the plating of 100 µL of appropriate dilutions on Middlebrook 7H10 plates and was followed by the calculation of CFU. The *Escherichia coli* and *Staphylococcus aureus* strains were cultured in Mueller-Hinton medium. The susceptibility of *E. coli* and *S. aureus* to the tested carbohydrates was determined by the microdilution method according to standard CLSI techniques [54]. The analysis was performed in 96-well microplates containing Mueller-Hinton broth. All of the tested compounds were dissolved in water or dimethyl sulfoxide (DMSO). The concentrations of the carbohydrates in Mueller-Hinton broth initially ranged from 10 to 0.078 mM. The DMSO control was included in each experiment. The inoculum density was adjusted to a 0.5 McFarland standard. The microplates were incubated at 37 °C for 18 h, and the OD_600_ measurements were determined for the bacterial cultures in the presence and absence of the tested compounds. The MIC analyses were repeated at least three times. 

### 4.5. In Vitro Cytotoxicity Assay

The cytotoxicity of the tested compounds was examined in vitro against mouse L929 fibroblasts. Briefly, the cells were seeded at 10^3^ cells per well in 96-well tissue culture plates and allowed to proliferate at 37 °C for 12 h. Confluent monolayers of fibroblasts were treated with different concentrations of the compounds up to 1000 µM in triplicate or replaced with fresh medium (untreated controls). The compounds were dissolved in dimethyl sulfoxide (DMSO) or water to form drug solutions and then were suspended in RPMI supplemented with 5% of FBS, 1% β-mercaptoethanol and antibiotics. The final concentration of DMSO in the medium was 0.1%. After a 16 h or 72 h incubation at 37 °C in 5% CO_2_, the number of viable cells was determined by the Cell Counting Kit 8 (CCK-8, Dojindo, Sigma, Munich, Germany). The 50% cytotoxic concentration (CC_50_) was defined as the concentration required to reduce the cell growth by 50% compared to the untreated controls and was calculated with BioDataFit 1.02 software (Chang Bioscience, Fremont, CA, USA).

### 4.6. Preparation of Human Monocyte-Derived Macrophages (MDMs)

Human monocytes were isolated from commercially available (Regional Blood Donation Station, Lodz, Poland) and freshly prepared buffy coats of healthy human blood donors using a double density gradient technique employing Histopaque-1077 (Sigma) and 46% iso-osmotic Percoll. In the first step of the procedure, the peripheral blood mononuclear cells (PBMC) were obtained. The buffy coat was diluted two times with Dulbecco’s phosphate buffered saline (Cytogen, Zgierz, Poland) without Ca^2+^ and Mg^2+^ and supplemented with 2 mM of EDTA (Sigma) (DPBS/EDTA) and was carefully layered onto Histopaque-1077 at a ratio of 2:1. The gradient was centrifuged at 400× *g* (without brake) and room temperature (RT) for 30 min. After centrifugation, the upper layer was gently discarded, and the opaque, white interface of the PBMC was transferred into a 50 mL clean conical tissue culture tube. The collected cells were washed three times with DPBS/EDTA (300× *g*, RT, 10 min), then the PBMC were resuspended in DPBS/EDTA again to remove platelets by low-speed centrifugation at 120× *g* (without brake) and RT for 10 min. An aliquot of the PBMC/DPBS/EDTA suspension was diluted 20-fold, and the number and viability (Trypan blue exclusion test) of the cells were determined using a haemocytometer (Bürker, VWR International, Radnor, PA, USA). Human monocytes were purified from PBMC using 46% iso-osmotic Percoll according to the previously described protocol [55] with a slight modification. Briefly, 25 mL of mononuclear cell suspension at a density of 5 × 10^6^/mL in Iscove’s medium (Cytogen) containing sodium bicarbonate, L-glutamine and HEPES, and supplemented with 10% foetal calf serum (Cytogen), (FCS), 100 U/mL penicillin and 100 µg/mL streptomycin (both antibiotics from Sigma) was layered onto an equal volume of the iso-osmotic gradient, and the human monocytes were separated by centrifugation at 550× *g* and RT for 30 min. The white band of the interface formed by the monocytes was collected and diluted with ice cold DPBS/EDTA, followed by centrifugation at 400× *g* and 4 °C for 10 min. The pellet of the human monocytes was suspended in DPBS, and the number and viability of the cells were estimated. Monocytes were differentiated into macrophages by culturing for 7 days in culture medium (Iscove’s medium with sodium bicarbonate, l-glutamine, HEPES, 10% Human AB serum, 0.005 mM 2-mercaptoethanol, 100 U/mL penicillin, and 100 µg/mL streptomycin) supplemented with 10 ng/mL of macrophage colony-stimulating factor (M-CSF) (Invitrogen) in 24-well tissue culture plates at a cell concentration of 5 × 10^5^/mL/well. The cells were incubated at 37 °C in a humidified atmosphere of 10% CO_2_/90% air. After 3–4 days of culturing, the monocytes were additionally fed using 0.5 mL of culture medium/M-CSF. To adequately calculate the number of monocytes plated into the wells, the cell suspension was examined morphologically under an optical microscope using Giemsa stained slides. The estimated purity of monocytes reached 80%.

### 4.7. Infection of MDMs with M. tuberculosis

Twenty-four hours before *Mtb* infection, the cultures of the differentiated human macrophages were extensively washed three times to remove any nonadherent cells: twice with Iscove’s medium supplemented with 2% human AB serum, 100 U/mL penicillin, and 100 µg/mL streptomycin and once with the same medium without antibiotics. Finally, 1 mL of the culture medium without antibiotics was added to each well, and the macrophages were left resting overnight. The next day, a suspension of *Mtb* (5 × 10^7^/mL) in culture medium without antibiotic was prepared, and the cells were infected with tubercle bacilli at an MOI of 1:10. Two hours after infection, extracellularly located bacteria were removed by thoroughly two times washing with the culture medium without antibiotics. To eliminate any unengulfed mycobacteria, 1 mL of culture medium supplemented with 50 µg/mL gentamicin (Sigma) was added to each well, and the infected macrophage cultures were incubated for one additional hour. Subsequently, the cells were washed three times with Iscove’s medium supplemented with 2% Human AB serum and were subjected to the experiments evaluating the bactericidal activity of the carbohydrates against the intracellularly growing tubercle bacilli.

### 4.8. Evaluation of the Bactericidal Effect of Carbohydrates against Intracellularly Growing Tubercle Bacilli

After killing the extracellularly located *Mtb* cells with gentamicin, the human phagocyte cultures were extensively washed, and the culture medium without antibiotics but supplemented with the carbohydrates was added to the independent samples of the infected macrophages. From the experiments on the mycobactericidal effect of the tested compounds against tubercle bacilli growing in broth cultures, the four carbohydrates, i.e., **6**, **16A**, **20** and **23**, exhibiting the highest antimycobacterial activity were selected for further determination of their bactericidal potential against intracellularly deposited *Mtb*. The chosen compounds were applied at the minimal inhibitory concentrations, namely, 50, 30, 30 and 25 µM, respectively. Each of the carbohydrates was tested in quadruplicate. The *Mtb*-infected macrophage cultures incubated with the culture medium alone (without antibiotics) served as controls. The experimental (carbohydrate-treated) and the control samples of the infected phagocytes were incubated at 37 °C for 48 h under a humidified atmosphere of 10% CO_2_/90% air. Subsequently, the macrophages were lysed with 1 mL of 0.1% SDS, and appropriate dilutions of the cell lysates were plated onto Middlebrook 7H10 agar supplemented with 10% of BBL™ Middlebrook oleic acid-albumin-dextrose-catalase (OADC) enrichment. After 14–21 days of growing, the number of CFU was counted. 

To exclude the influence of the cytotoxicity of carbohydrates **6**, **16A**, **20**, and **23** on the results of the performed experiments, the uninfected macrophages were treated with the same concentrations of the tested compounds and cultured under the aforementioned conditions. Simultaneously, the controls, untreated samples of human phagocytes, were incubated. After 48 h of incubation, MTT ((3-(4,5-dimethylthiazol-2-yl)-2,5-diphenyltetrazolium bromide), Sigma) at a concentration of 5 mg/mL in DPBS was added to each well at a ratio of 1:10. The conversion of soluble MTT to water-insoluble formazan crystals by the macrophages was carried out for an additional 4 h under the same conditions, followed by centrifugation at 583× *g* for 5 min. The supernatants were discarded, and the formazan crystals were dissolved with 750 µL DMSO and 125 µL glycine buffer (pH 10.5). The absorbance values (OD) were measured at λ = 570 nm with a Multiscan EX automatic ELISA reader (Thermo Scientific, FI-01621-Vantaa, Finland). All experimental and control samples were run in quadruplicate.

### 4.9. Statistical Analysis

The one-way analysis of variance (ANOVA) (Holm-Sidak method) was employed for multiple comparisons versus the control samples to determine any significant differences between the mean values of the carbohydrate-untreated and carbohydrate-treated samples of both the *Mtb*-infected and *Mtb*–uninfected human macrophages. The results were considered to be statistically significant at *p* < 0.05.

## Figures and Tables

**Table 1:** The bactericidal effect of the thio- functionalized carbohydrate derivatives.

Compounds	Tested Anti-*Mtb*	Tested Anti-*E. coli*, *S. aureus*
5-thio-d-glucose pentaacetate (**1**)	MIC_50_ > 1 mM/OD	ND
6-thio-β-d-fructopyranose (**2**)	MIC_50_ > 1 mM/OD	MIC_50_ > 2 mM/*E. coli* MIC_50_ > 2 mM/*S. aureus*
1,6-anhydro-5-*C*-hydroxymethyl-α-l-altro-pyranose (**3**)	MIC_50_ > 1 mM/OD	ND
2-*C*-methylene-myo-inositol oxide (**4**)	MIC_50_ > 1 mM/OD	ND
1,5-anhydro-6-deoxy-6-methanesulfamido-d-glucitol (**5**)	MIC_50_ > 1 mM/OD	MIC_50_ > 2 mM/*E. coli* MIC_50_ > 2 mM/*S. aureus*
1,6-anhydro-3-deoxy-4-*S*-(2,3,4,6-tetra-*O*-acetyl-β-d-glucopyranosyl-d-glycero-*hexopyranos*-2-ulose (**6**)	MIC_50_ = 0.05 mM/CFU	MIC_50_ = 5 mM/*E. coli* MIC_50_ = 2.5 mM/*S. aureus*
1,6-anhydro-3-deoxy-4-S-β-d-glucopyranosyl-d-glycero-*hexopyranos*-2-ulose (**7**)	MIC_50_ = 0.5 mM/CFU	MIC_50_ > 10 mM/*E. coli* MIC_50_ > 10 mM/*S. aureus*
1,6-anhydro-3-deoxy-4-*S*-β-salicylyl-d-glycero-*hexopyranos*-2-ulose (**8**)	MIC_50_ = 0.2 mM/CFU	MIC_50_ > 10 mM/*E. coli* MIC_50_ > 10 mM/*S. aureus*
1,6-anhydro-3-deoxy-4-*S*-(2,3,4,6-tetra-*O*-acetyl-β-d-galactopyranosyl-d-glycero-*hexopyranos*-2-ulose (**9**)	MIC_50_ > 0.15 mM/OD	ND
2-acetamido-2-dexy-1,5-anhydro-d-mannitol (**10**)	MIC_50_ > 1 mM/OD	MIC_50_ > 10 mM/*E. coli* MIC_50_ > 10 mM/*S. aureus*
1,2-*O*-isopropylidene-5,6-anhydro-d-glucofuranose (**11**)	MIC_50_ = 0.75 mM/OD	ND
1,2-*O*-isopropylidene-3,5-di-*O*-acetyl-6-methoxy-d-glucofuranose (**12**)	MIC_50_ > 1 mM/OD	MIC_50_ > 5 mM/*E. coli* MIC_50_ > 10 mM/*S. aureus*
1-*C*-nitromethyl-d-gluco-pyranose (**13**)	MIC_50_ > 1 mM/OD	MIC_50_ > 10 mM/*E. coli* MIC_50_ > 10 mM/*S. aureus*
1,6-anhydro-3,4-dideoxy-α-d-glycero-*hex-3-enopyranos*-2-thiosemicarbazone (**14**)	MIC_50_ = 0.35 mM/OD	MIC_50_ > 5 mM*/E. coli* MIC_50_ > 5 mM*/S. aureus*
1,6-anhydro-3-deoxy-4-*S*-(octa-*O*-acetyl-β-d-lactosyl-d-glycero-*hexopyranos*-2-ulose (**16**)	MIC_50_ = 0.5 mM/OD	MIC_50_ > 5 mM*/E. coli* MIC_50_ > 5 mM*/S. aureus*
octa-*O*-acetyl-β-d-lactosyl-*S*-1-adamantane (**19**)	MIC_50_ = 0.5 mM/OD	ND
1,6-anhydro-3,4-dideoxy-α-d-glycero-*hexopyranos*-2-thiosemicarbazone (**14A**)	MIC_50_ > 1 mM/OD	MIC_50_ > 2.5 mM/*E. coli* MIC_50_ > 1.25 mM/*S. aureus*
1,6-anhydro-3-deoxy-4-*S*-(octa-*O*-acetyl-β-d-lactosyl-d-glycero-*hexopyranos*-2-thiosemicarbazone (**16A**)	MIC_50_ = 0.03 mM/CFU	MIC_50_ > 150 µM/*E. coli* MIC_50_ > 1.25 mM/*S. aureus*
1,6-anhydro-3-deoxy-4-*S*-(2,3,4-tri-*O*-acetyl-β-l-arabino)-d-glycero-*hexopyranos*-2-ulose (**20**)	MIC_50_ = 0.03 mM/CFU	MIC_50_ > 300 µM/*E. coli* MIC_50_ > 1.25 mM/*S. aureus*
1,6-anhydro-3-deoxy-4-*S*-(5-amino-1,3,4-thiadiazol-2-yl)-d-glycero-*hexopyranos*-2-ulose (**23**)	MIC_50_ = 0.025 mM/CFU	MIC_50_ > 1.25 mM/*E. coli* MIC_50_ > 0.625 mM/*S. aureus*
1,6-anhydro-3-deoxy-4-*S*-(4,5-dihydrothiazol-2-yl-d-glycero-*hexopyranos*-2-ulose (**26**)	MIC_50_ = 0.05 mM/CFU	MIC_50_ > 2.5 mM/*E. coli* MIC_50_ > 2.5 mM/*S. aureus*

ND—not determined.

**Table 2:** Cytotoxic effect of carbohydrates **6**, **16A**, **20** and **23** on the human monocyte-derived macrophages (MDMs).

Experimental Sample	OD_[570nm]_ ± SD	Cell Viability [%]
MDMs/medium	2.381 ± 0.038	n/a
MDMs/6	2.163 ± 0.054	91
MDMs/16A	2.345 ± 0.043	98
MDMs/20	2.269 ± 0.048	95
MDMs/23	2.428 ± 0.031	100

**Table 3:** IC_50_ values for mice fibroblasts.

Compounds	IC_50_ [µM]
**1**	>1000
**2**	>1000
**6**	45
**7**	504
**8**	515
**9**	217
**14**	329
**14a**	>1000
**16**	250
**16a**	240
**19**	227
**20**	500
**23**	4
**26**	11

**Table 4:** The thio-functionalized carbohydrate derivatives used in this study.

Compound	LogP/cLog	References
5-thio-D-glucose pentaacetate (**1**)	LogP: −0.51 cLog: 0.72825	[19]
6-thio-β-d-fructopyranose (**2**)	LogP: −1.36 cLog: −1.9984	[19]
1,6-anhydro-5-*C*-hydroxymethyl-α-l-altro-pyranose (**3**)	LogP: −2.38 cLog: −1.5973	[19]
2-*C*-methylene-myo-inositol oxide (**4**)	LogP: −3.22 cLog: −11214	[19]
1,5-anhydro-6-deoxy-6-methanesulfamido-d-glucitol (**5**)	LogP: −3.6 cLog: −1.1515	[19]
1,6-anhydro-3-deoxy-4-*S*-(2,3,4,6-tetra-*O*-acetyl-β-d-glucopyranosyl-d-glycero-*hexopyranos*-2-ulose (**6**)	LogP: −1.13 cLog: 0.4407	[18,42]
1,6-anhydro-3-deoxy-4-*S*-β-d-glucopyranosyl-d-glycero-*hexopyranos*-2-ulose (**7**)	LogP: −2.05 cLog: −1.69975	[18,42]
1,6-anhydro-3-deoxy-4-*S*-β-salicylyl-d-glycero-*hexopyranos*-2-ulose (**8**)	LogP: 1.28 cLog: 0.8949	[43]
1,6-anhydro-3-deoxy-4-*S*-(2,3,4,6-tetra-*O*-acetyl-β-d-galactopyranosyl-d-glycero-*hexopyranos*-2-ulose (**9**)	LogP: −1.13 cLog: 0.4407	[18,42]
2-acetamido-2-dexy-1,5-anhydro-d-mannitol (**10**)	Log P: −2.75 cLog: 0.993001	[44]
1,2-*O*-isopropylidene-5,6-anhydro-3-hydroxy-d-glucofuranose (**11**)	LogP: −0.39 cLog: 0.64599	[45]
1,2-*O*-isopropylidene-3,5-di-*O*-acetyl-6-methoxy-d-glucofuranose (**12**)	LogP: −0.2 cLog: 0.9672	[46]
1-C-nitromethyl-d-gluco-pyranose (**13**)	LogP: −4.22 cLog: −1.9428	[47]
1,6-anhydro-3,4-dideoxy-α-d-glycero-*hex-3-enopyranos*-2-thiosemicarbazone (**14**)	LogP:−0.18 cLog:0.372	[48,49]
1,6-anhydro-3-deoxy-4-*S*-(octa-*O*-acetyl-β-d-lactosyl-d-glycero-*hexopyranos*-2-ulose (**16**)	LogP:−2.18 cLog:0.854498	[48,49]
1,6-anhydro-3,4-dideoxy-α-d-glycero-*hexopyranos*-2-thiosemicarbazone (**14A**)	LogP: −0.29 cLog: 0.204	[48,49]
1,6-anhydro-3-deoxy-4-*S*-(octa-*O*-acetyl-β-d-lactosyl-d-glycero-*hexopyranos*-2-thiosemicarbazone (**16A**)	LogP: NA cLog: 1.26	[48,49]
Octa-*O*-acetyl-β-d-lactosyl-*S*-1-adamantane (**19**)	cLog: 4.0538	[49]
1,6-anhydro-3-deoxy-4-*S*-(2,3,4-tri-*O*-acetyl-β-l-arabino)-d-glycero-*hexopyranos*-2-ulose (**20**)	LogP: −0.9 cLog: 0.632201	[49]
1,6-anhydro-3-deoxy-4-*S*-(5-amino-1,3,4-thiadiazol-2-yl)-d-glycero-*hexopyranos*-2-ulose (**23**)	LogP: 1.37 cLog: −0.535599	[50] & this work
1,6-anhydro-3-deoxy-4-*S*-(4,5-dihydrothiazol-2-yl-d-glycero-*hexopyranos*-2-ulose (**26**)	LogP: 1.13 cLog: 0.0390999	[50] & this work

## Supplementary Materials

Supplementary materials are available online.
