# Supplementary material for: Evaluation of the Mycobactericidal Effect of Thio-functionalized Carbohydrate Derivatives
Source: Molecules. 2017 May 16;22(5):812. doi: 10.3390/molecules22050812 (PMC6154314; doi:10.3390/molecules22050812)

Supplementary Materials

Evaluation of the bactericidal effect of thio-functionalized carbohydrate derivatives.

Małgorzata Korycka-Machała[1], Anna Brzostek[1], Bożena Dziadek[2], Malwina Kawka[2], Tomasz Popławski[3], Zbigniew J. Witczak[4]# and Jarosław Dziadek[1]#

AM_Reaction_23_22Mar17

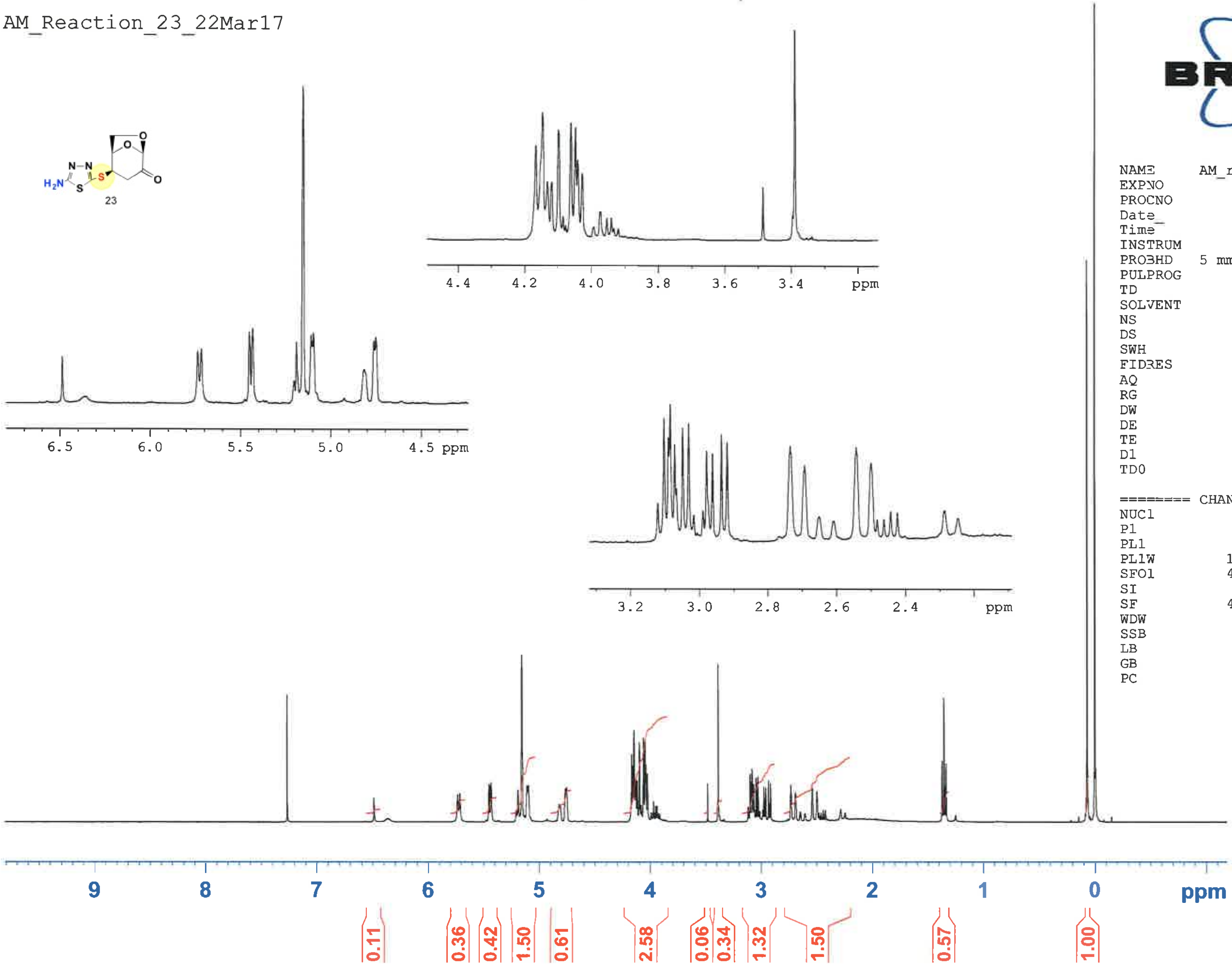

NAME	AM_reaction23_22Mar17
EXPNO	10
PROCNO	1
Date_	20170322
Time_	11.43
INSTRUM	spect
PROBHD	5 mm PABBO BB-
PULPROG	zg30
TD	65536
SOLVENT	CDCl3
NS	64
DS	2
SWH	8223.685 Hz
FIDRES	0.125483 Hz
AQ	3.9846387 sec
RG	203
DW	60.800 usec
DE	6.50 usec
TE	293.7 K
D1	1.00000000 sec
TD0	1

======== CHANNEL f1 ========

NUC1	1H
P1	14.50 usec
PL1	-1.50 dB
PL1W	12.52491283 W
SFO1	400.1324710 MHz
SI	32768
SF	400.1300062 MHz
WDW	EM
SSB	0
LB	0.30 Hz
GB	0
PC	1.00

AM_Reaction_23_22Mar17

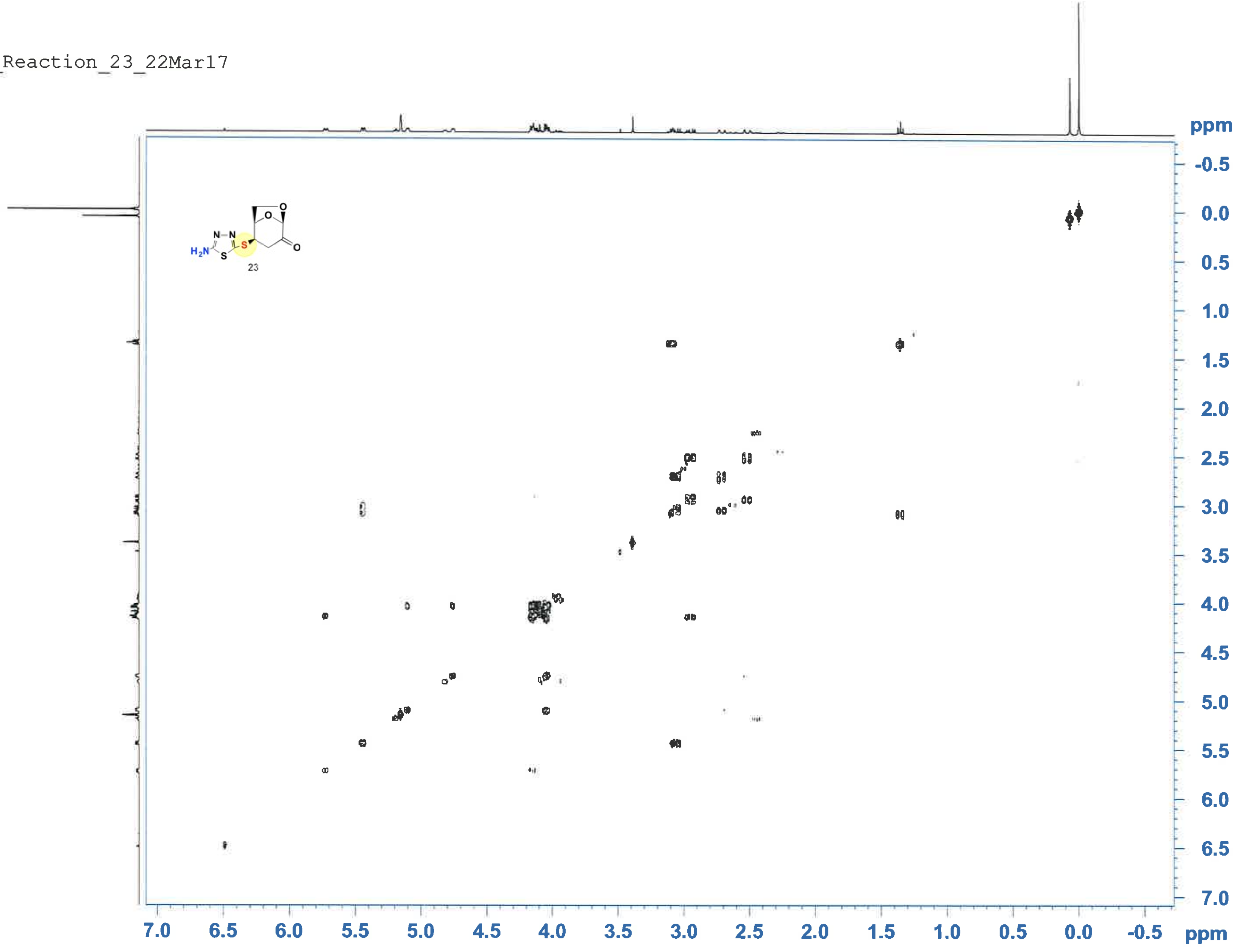

AM_Reaction_23_22Mar17

23

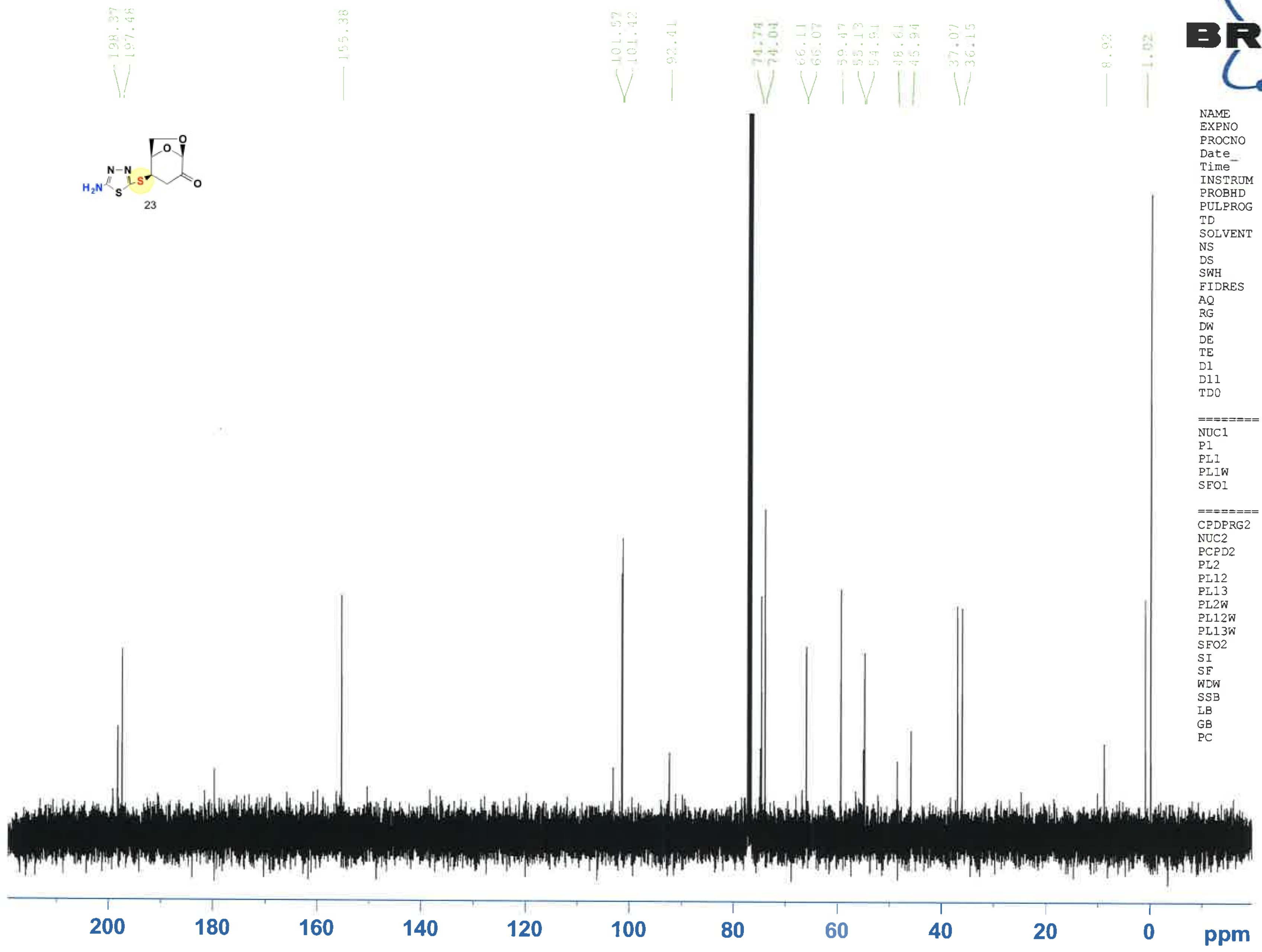

NAME	AM_reaction23_22Mar17
EXPNO	12
PROCNO	1
Date_	20170322
Time	12.04
INSTRUM	spect
PROBHD	5 mm PABBO BB-
PULPROG	zgpg30
TD	65536
SOLVENT	CDCl3
NS	256
DS	4
SWH	24038.461 Hz
FIDRES	0.366798 Hz
AQ	1.3631988 sec
RG	203
DW	20.800 usec
DE	6.50 usec
TE	295.6 K
D1	2.00000000 sec
D11	0.03000000 sec
TD0	1

======== CHANNEL f1 ========

NUC1	13C
P1	8.80 usec
PL1	-3.00 dB
PL1W	66.65790558 W
SFO1	100.6228298 MHz

======== CHANNEL f2 ========

CPDPRG2	waltz16
NUC2	1H
PCPD2	80.00 usec
PL2	-1.50 dB
PL12	13.33 dB
PL13	13.00 dB
PL2W	12.52491283 W
PL12W	0.41188380 W
PL13W	0.44440067 W
SFO2	400.1316005 MHz
SI	32768
SF	100.6127690 MHz
WDW	no
SSB	0
LB	0.00 Hz
GB	0
PC	1.40

AM Reaction 23 22Mar17

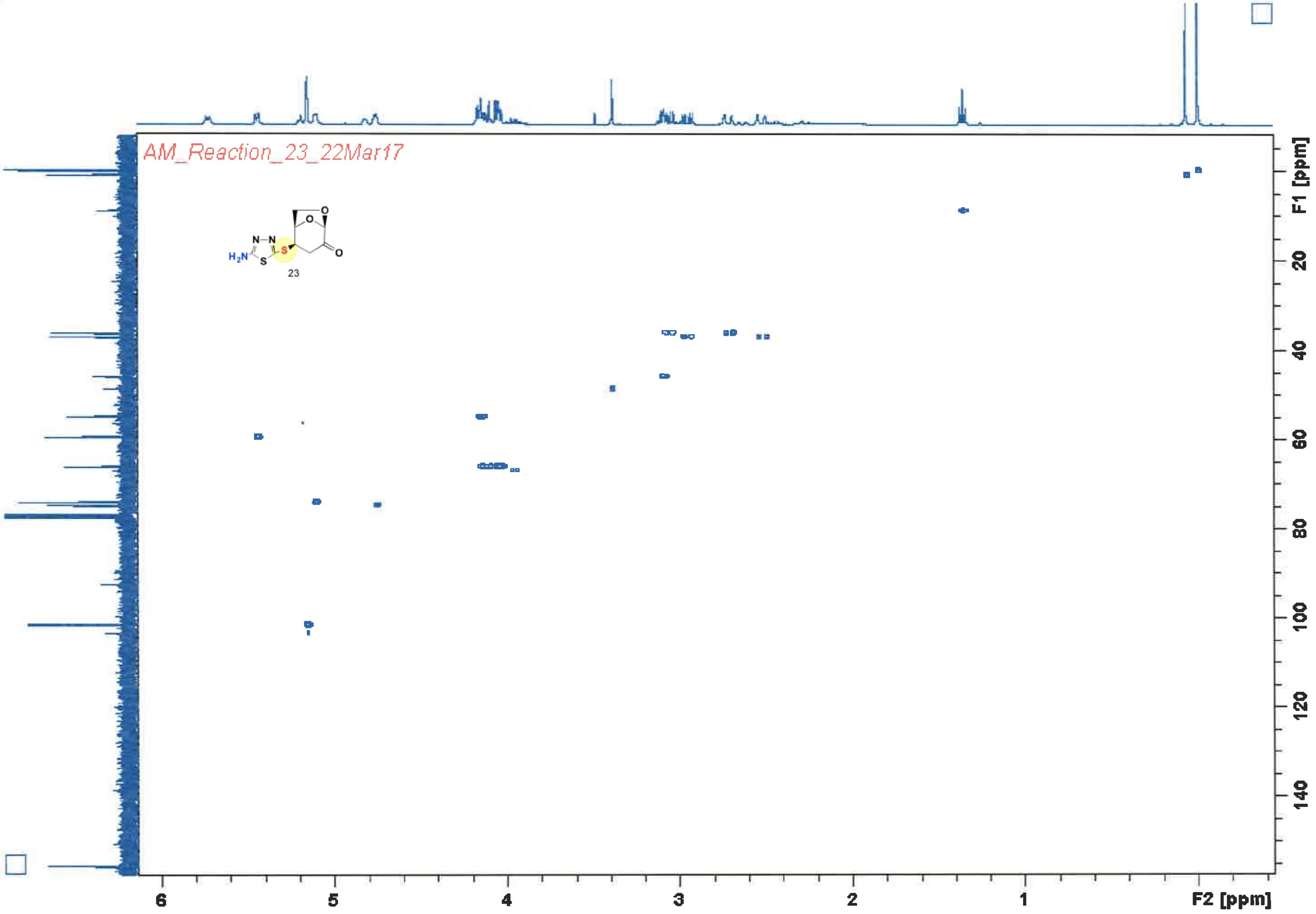

AM_Reaction_26_22Mar17

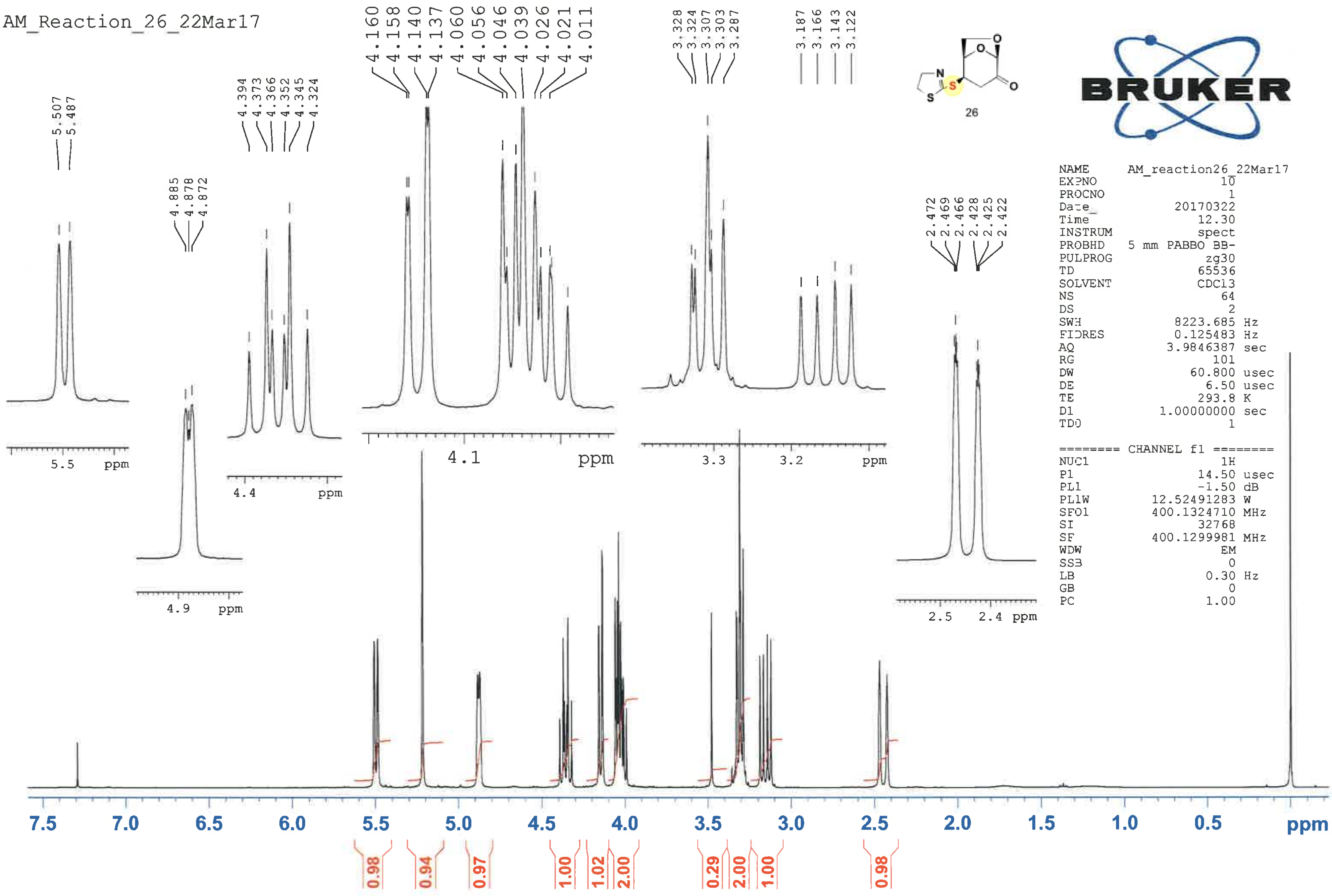

AM_Reaction_26_22Mar17

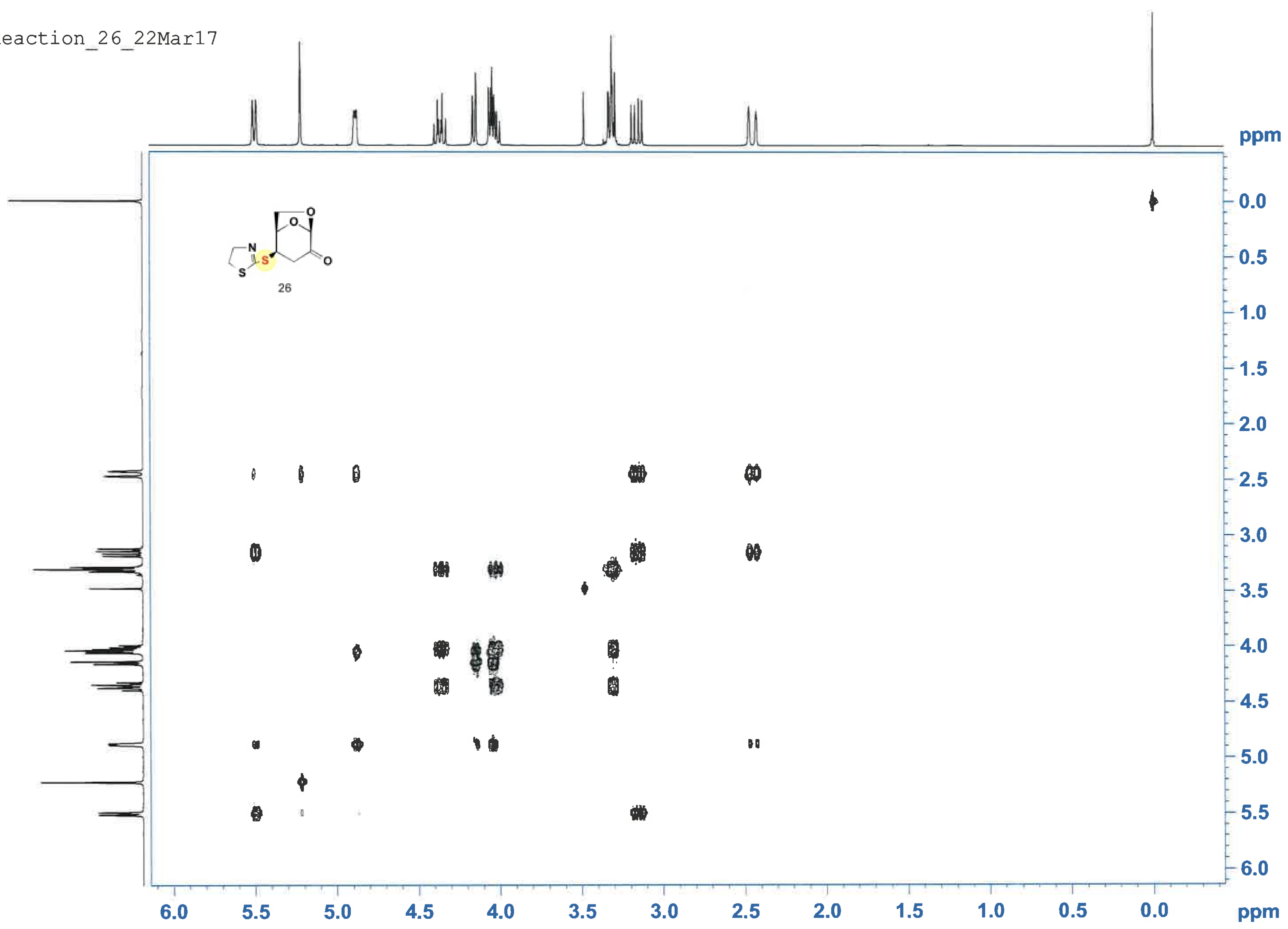

AM_Reaction_26_22Mar17

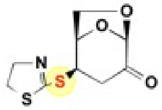

26

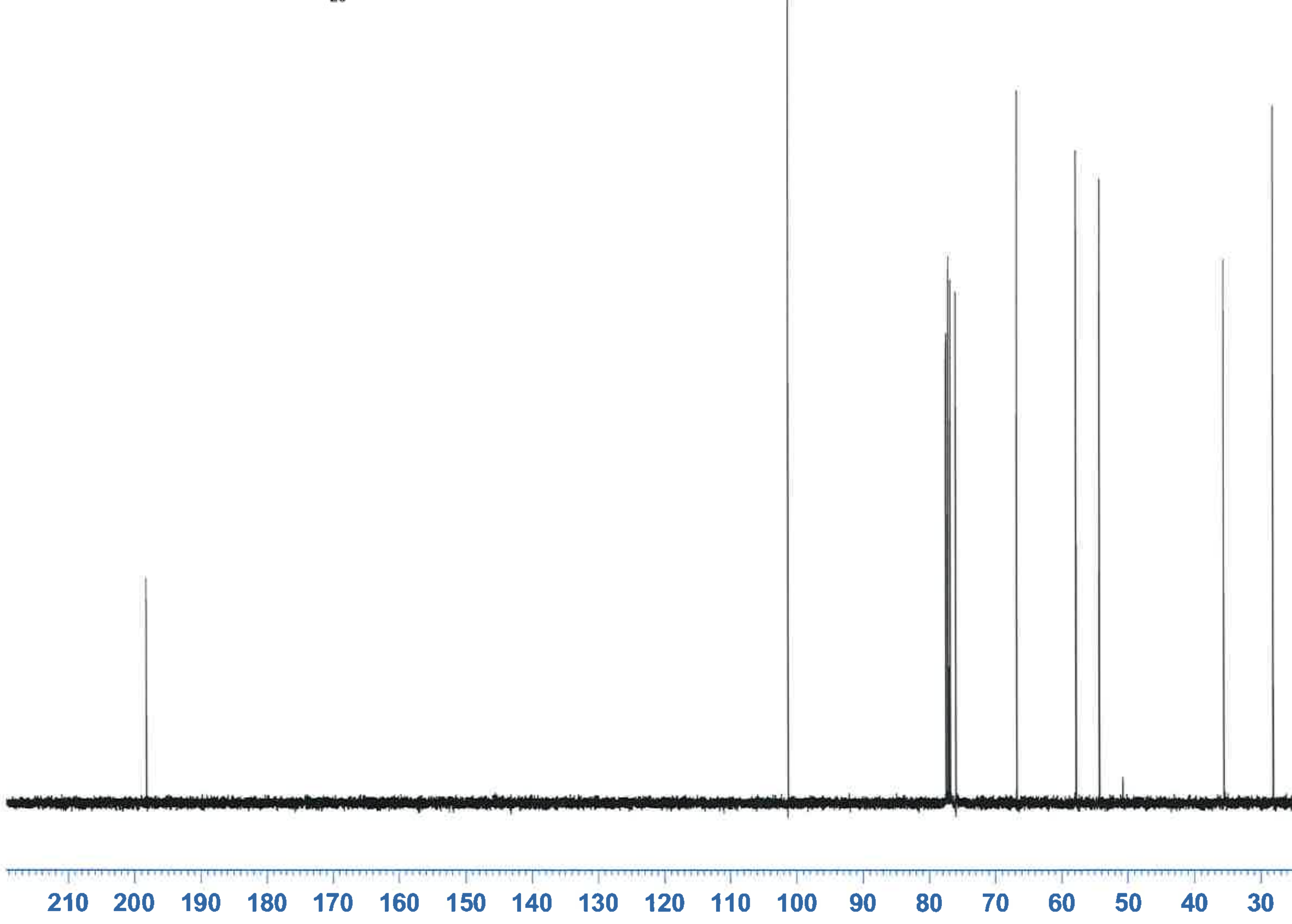

BRUKER

NAME	AM_reaction26_22Mar17	
EXPNO	12	
PROCNO	1	
Date_	20170322	
Time	12.51	
INSTRUM	spect	
PROBHD	5 mm PABBO BB-	
PULPROG	zgpg30	
TD	65536	
SOLVENT	CDCl3	
NS	256	
DS	4	
SWH	24038.461	Hz
FIDRES	0.366798	Hz
AQ	1.3631988	sec
RG	203	
DW	20.800	usec
DE	6.50	usec
TE	295.6	K
D1	2.00000000	sec
D11	0.03000000	sec
TD0	1	
========	CHANNEL f1	========
NUC1	13C	
P1	8.80	usec
PL1	-3.00	dB
PL1W	66.65790558	W
SFO1	100.6228298	MHz
========	CHANNEL f2	========
CPDPRG2	waltz16	
NUC2	1H	
PCPD2	80.00	usec
PL2	-1.50	dB
PL12	13.33	dB
PL13	13.00	dB
PL2W	12.52491283	W
PL12W	0.41188380	W
PL13W	0.44440067	W
SFO2	400.1316005	MHz
SI	32768	
SF	100.6127690	MHz
WDW	no	
SSB	0	
LB	0.00	Hz
GB	0	
PC	1.40	

AM_Reaction_26_22Mar17

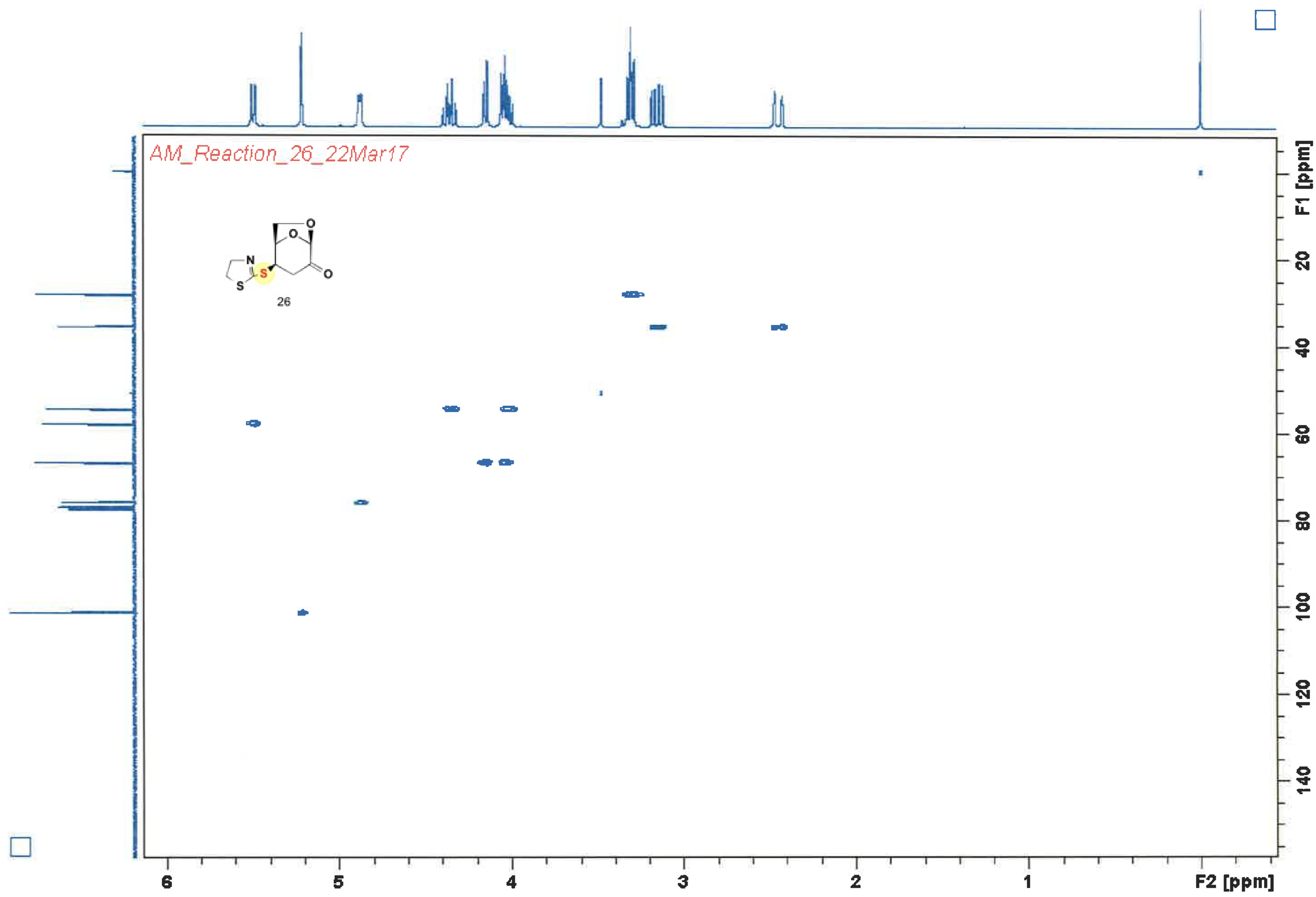

Supplement: Supplementary file 1 [file molecules-22-00812-s001.pdf]
